# Health Care Resource Use and Total Mortality After Hospital Admission for Severe COVID-19 Infections During the Initial Pandemic Wave in France: Descriptive Study

**DOI:** 10.2196/56398

**Published:** 2024-09-11

**Authors:** Mikhail Dziadzko, Manon Belhassen, Eric Van Ganse, Fabrice Heritier, Marjorie Berard, Claire Marant-Micallef, Frederic Aubrun

**Affiliations:** 1 Hospices Civils de Lyon, Hôpital de la Croix Rousse, Département d’Anesthésie-Réanimation, Douleur Lyon France; 2 Laboratoire RESHAPE, Université Claude Bernard Lyon 1, INSERM UMR 1290 Lyon France; 3 PELyon Lyon France; 4 Hospices Civils de Lyon, Hôpital de la Croix Rousse, Département de la Médecine Respiratoire Lyon France; 5 Centre Hospitalier de Roanne, Département d’Anesthésie-Réanimation Roanne France

**Keywords:** claims data, COVID-19 infection, France, health care resource utilizationuse, hospitalization, mortality, Post-Acute COVID-19 Syndrome, PACS, analysis, COVID-19, health care, infection, infections, pandemic, descriptive study, resource use, outpatient care, retrospective, cohort study, women, female, females, population-based

## Abstract

**Background:**

Little is known about post–hospital health care resource use (HRU) of patients admitted for severe COVID-19, specifically for the care of patients with postacute COVID-19 syndrome (PACS).

**Objective:**

A list of HRU domains and items potentially related to PACS was defined, and potential PACS-related HRU (PPRH) was compared between the pre– and post–COVID-19 periods, to identify new outpatient care likely related to PACS.

**Methods:**

A retrospective cohort study was conducted with the French National Health System claims data (SNDS). All patients hospitalized for COVID-19 between February 1, 2020, and June 30, 2020 were described and investigated for 6 months, using discharge date as index date. Patients who died during index stay or within 30 days after discharge were excluded. PPRH was assessed over the 5 months from day 31 after index date to end of follow-up, that is, for the post–COVID-19 period. For each patient, a pre–COVID-19 period was defined that covered the same calendar time in 2019, and pre–COVID-19 PPRH was assessed. Post- or pre- ratios (PP ratios) of the percentage of users were computed with their 95% CIs, and PP ratios>1.2 were considered as “major HRU change.”

**Results:**

The final study population included 68,822 patients (median age 64.8 years, 47% women, median follow-up duration 179.3 days). Altogether, 23% of the patients admitted due to severe COVID-19 died during the hospital stay or within the 6 months following discharge. A total of 8 HRU domains were selected to study PPRH: medical visits, technical procedures, dispensed medications, biological analyses, oxygen therapy, rehabilitation, rehospitalizations, and nurse visits. PPRs showed novel outpatient care in all domains and in most items, without specificity, with the highest ratios observed for the care of thoracic conditions.

**Conclusions:**

Patients hospitalized for severe COVID-19 during the initial pandemic wave had high morbi-mortality. The analysis of HRU domains and items most likely to be related to PACS showed that new care was commonly initiated after discharge but with no specificity, potentially suggesting that any impact of PACS was part of the overall high HRU of this population after hospital discharge. These purely descriptive results need to be completed with methods for controlling for confusion bias through subgroup analyses.

**Trial Registration:**

ClinicalTrials.gov NCT05073328; https://clinicaltrials.gov/ct2/show/NCT05073328

## Introduction

The burden of COVID-19 pandemic has been high in all countries [1,2]. In France, more than 200,000 patients have been hospitalized for severe COVID-19 infections in 2020, and 20% of them deceased [3]. Hospitalized patients with COVID-19 generated a high level of health care resource use (HRU) during hospitalization, with more than one-third of inpatients also requiring specific health care services after discharge [4].

Persistent debilitating symptoms (including fatigue, breathlessness, and muscle and joint pain) were previously described in more than 30% of infections, and a new condition, long-COVID, or postacute COVID-19 syndrome (PACS) has been identified in more than 50% of those who were hospitalized for COVID-19 [5-7].

Besides nonspecific complaints, PACS also commonly includes cognitive disorders and psychological symptoms, with anxiety, depression and posttraumatic stress symptoms being described [8]. Anecdotal data suggest that COVID-19 did significantly and nonspecifically impact the overall health status of infected patients and their HRU, with an increased demand for primary health care providers and private practice physicians’ consultations, as well as outpatient care resource use. For instance, according to the phone call–based population sample French APCOVID-19 study, patients with PACS generated a 10% increase in family physician and private practice specialists, compared to patients without PACS [9]. In parallel, severe COVID-19 infections are known to have affected preexisting chronic conditions, likely leading to increased burden of these conditions, such as congestive heart failure or diabetes [10,11].

Since acute COVID-19 infections may affect the overall health status, it would be of interest to assess their specific impact on the HRU of infected patients, to distinguish new, acute, COVID-19–induced conditions—such as new onset pain—from chronic conditions possibly affected by COVID-19—such as worsened diabetes. This would help identify PACS-related HRU, so enabling the allocation of specific, and appropriate, resources to the care of this newly described condition.

A tool is available in France to assess HRU in the overall population: the National Healthcare Data System (Système National des Données de Santé [SNDS]) that has been developed since 2007 for all reimbursed cares of more than 98% of the French population, both in primary and secondary care [12].

The primary objective of our study was to define and assess the potential PACS-related HRU (PP Ratio HRU) in patients discharged from hospital after severe COVID-19, and to assess how PP Ratio HRU changed compared to its prepandemic figures, from the assumption that the impact of PACS can be assessed from specific HRU data.

## Methods

### Data Source

This retrospective, population-based cohort study was based on the French National Health System claims database (SNDS). It contains anonymous individual information on sociodemographic characteristics, all nonhospital reimbursed health care expenditures (without corresponding medical diagnoses), and all hospital discharge summaries (*ICD-10* [*International Statistical Classification of Diseases, Tenth Revision*] code–based). The SNDS does not provide information on behavioral or clinical baseline characteristics (such as tobacco use, BMI, or diet or physical activity), nor biological or technical results. This claims database currently covers more than 98% of the country’s population [12].

### Study Design, Population, and Periods

This was a retrospective population-based cohort study. The study population consisted of all patients hospitalized for COVID-19 (*ICD-10* codes U07.10, U07.11, U07.14, U07.15 as a main diagnosis) between February 1, 2020, and June 30, 2020. The discharge date was defined as the study index date. Patients deceased during the hospitalization or in the 30 days following discharge, those with a pregnancy identified between February 1, 2019, and December 31, 2020, and those without any HRU in the 12 months before index date were excluded. Patients were followed up until death or for a maximum of 6 months, that is, until December 31, 2020, at the latest. The 5-month period starting at day 31 after the index date was defined as the post–COVID-19 period, and the corresponding 5-month period that occurred 12 months before the post–COVID-19 period (ie, in 2019) was defined as the pre–COVID-19 period.

For the patients included in the final cohort, age at index date, gender, and Charlson comorbidity index were extracted or computed from the SNDS data [13].

**Figure 1:**
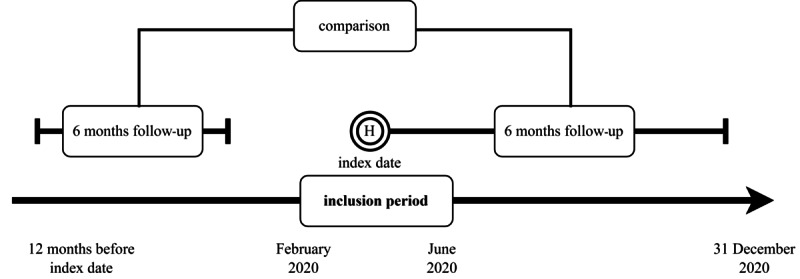
The study design and timeline for analyzing health care resource use among patients with COVID-19 during the first pandemic wave in France between February 1 and June 30, 2020. The figure illustrates a discharge date (index date), a 30-day postdischarge period, and the main study periods: 5-month pre– and post–COVID-19 periods for data collection regarding health care resource use. This pre-post retrospective design allows one to distinguish between new, acute COVID-19–induced conditions and exacerbations of preexisting chronic conditions, aiming to quantify and characterize the health care impact of severe COVID-19 infection beyond the initial hospitalization.

The HRU of selected patients was retrieved between the start of the second month (day 31) after discharge until the end of the follow-up—either until death or 6 months after the discharge, that is, the early post–COVID-19 period. To study COVID-19–induced changes, the HRU of the patients was also quantified over the pre–COVID-19 period (Figure 1).

### Outcome Variables

At the time of study initiation, the relevant evidence was synthetized by identifying the most frequent symptoms associated with PACS, that is, the symptoms of interest (SOIs) [14-17]. Next, based on the French Health Authority recommendations effective at the time of study conception [17] and with the support of expert clinicians (BLOC Study Group) involved in COVID-19 care in France, the domains of HRU that are potentially linked with SOIs management were selected. Within each domain, reimbursed acts potentially relevant to PACS management were next selected, and subsequently retrieved and investigated for the post– and pre–COVID-19 periods, to identify interventions newly introduced after hospital discharge.

### Statistical Analysis

For each item of all domains of interest, the number and percentage of patients using one or more items of the domain during the study period were extracted. A post- or pre- ratio (PP ratio) was then defined for each domain and for all items, as the ratio of the post–COVID-19 percentage of users divided by the pre–COVID-19 percentage of users. The HRU rate was calculated as a number of HRU uses per month and in the same patients for post– and pre–COVID-19 hospitalization study periods.

To address the potential underestimation of HRU increases due to counting HRU only once per patient, we calculated a post-pre ratio rate of HRU for each item with 95% CI using the mean HRU rates, standard deviations, and sample sizes for the periods before and after index hospitalization. This method uses log transformation to stabilize variance and ensure that the confidence interval remains on a proper scale when exponentiated back (see Multimedia Appendix 1). The HRU rate ratio provides information on the relative change in HRU frequency between the pre– and post–COVID-19 periods indicating the increase (PP Ratio>1) or decrease (<1) in the number of patients with HRU or the frequency of HRU use, especially for medical visits and dispensed medications, as the majority of French residents have high rates of baseline use of these resources [18,19].

Arbitrarily, the major changes in HRU occurred if 2 conditions were simultaneously met: (1) the post–COVID-19 percentage of patients using the domain or item was ≥1% of the study population; (2) the PP ratio of the percentages of the domain or item was ≥1.2.

Data are presented as frequencies and percentages; means and standard deviation; medians and IQR, and means or absolute numbers with 95% CI. All statistical analyses were performed using SAS (version 9.4; SAS Institute).

### Ethical Considerations

This study was conducted using the French National Health System claims database via the platform “French Health Data Hub” [20] and was approved by an independent French Scientific and Ethical Committee for Research, Studies, and Evaluations in the Health Sector (Comité éthique et scientifique pour les recherches, les études et les évaluations dans le domaine de la santé [CESREES]; 4653731, dated July 8, 2021). The committee authorized access to the data for the research team. The research project did not involve human participants; it was conducted using retrospective, fully anonymized data, and was approved by the National Informatics and Liberty Committee (CNIL, 921290, dated July 19, 2021). All calculations were performed remotely on the Health Data Hub platform; therefore, no Institutional Review Board approval was required at any stage of the research. The paper follows the Strengthening the Reporting of Observational Studies in Epidemiology (STROBE) guidelines [21].

## Results

### Study Population

**Figure 2:**
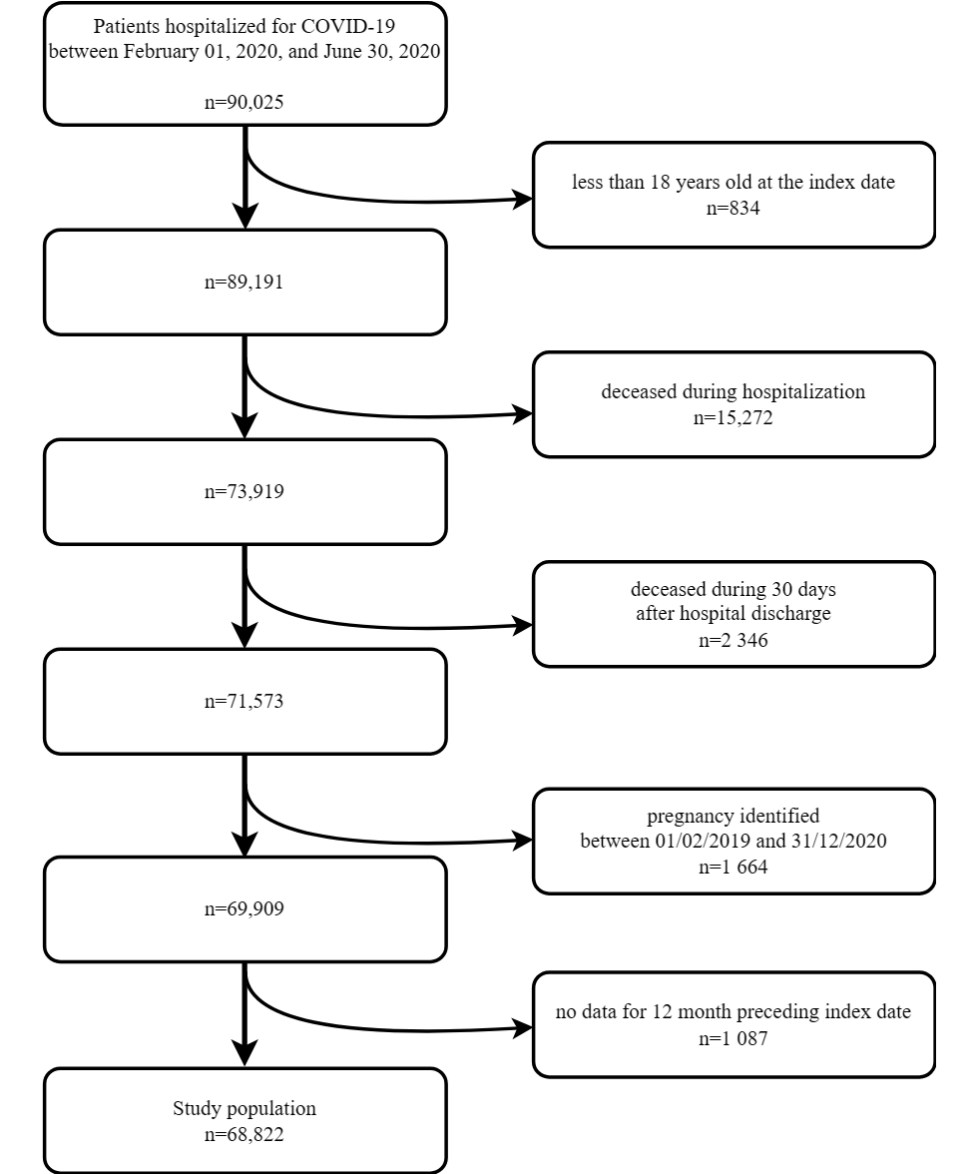
Study flowchart illustrating the patient selection process. Starting with an initial population of 90,025 patients hospitalized with a main diagnostic code of COVID-19 between February 1 and June 30, 2020, the figure shows the sequential application of exclusion criteria leading to final study population of 68,822 patients. This selection process ensures that the study focuses on survivors of the acute phase of COVID-19 with a history of health care use, allowing for meaningful comparison of pre– and post–COVID-19 health care resource use patterns.

Between February 1, 2020, and June 30, 2020, a total of 90,025 patients were hospitalized with a main diagnostic code of COVID-19. After applying successive exclusion criteria, the final study population comprised 68,822 patients (Figure 2 and Table 1).

The participants’ median age was 66 (18-108) years, and 32,372 (47%) were women. The median follow-up duration was 183 (31 to 184) days. A total of 17,618 patients deceased during the hospital stay or the 30 days following discharge, that is, 19.6% of the initial population. An additional 3117 patients died between the start of the second month post discharge and the end of the follow-up, bringing the total percentage of patients deceased to 23% of the initial population admitted for severe COVID-19.

**Table 1:** Characteristics of the study population with key characteristics of 68,822 survivors of COVID-19, including demographics, hospitalization details, and prevalent comorbidities.

Characteristics of patients		Values
Age (years), mean (SD)		64.8 (18.2)
**Age proportions (years), n (%)**		
	<65	32,670 (47.5)
	≥65	36,152 (52.5)
Female, n (%)		32,372 (47)
Follow up duration (days), mean (SD)		179.3 (20.9)
Insurance status (CMU-C^a^), n (%)		4923 (15.1)
Charlson score, mean (SD)		3.6 (3.2)
LOS^b^ for index hospitalization (days), mean (SD)		12.0 (8)
Admitted to the ICU^c^, n (%)		8646 (12.6)
Admitted to step down unit, n (%)		7175 (10.4)
Admitted to high dependency unit, n (%)		930 (14)
SAPS II^d^, mean (SD)		34.0 (13.4)
Oxygen therapy or NIV^e^ during index hospitalization, n (%)		13,199 (19.2)
Invasive ventilation during index hospitalization, n (%)		5820 (8.5)
Discharge at home, n (%)		53,092 (77.1)
**Comorbidities, n (%)**		
	Diabetes	16,029 (23.3)
	Stroke	4112 (6.0)
	Vascular diseases	5256 (7.6)
	Cardiac insufficiency	16,223 (23.6)
	Coronary disease	8058 (11.7)
	Respiratory insufficiency	6518 (9.5)
	Cystic fibrosis	26 (<0.1)
	Chronic kidney disease	6688 (9.7)
	Psychiatric disorders	12,066 (17.5)
	Active tuberculosis	104 (0.2)
	Malignancy	9655 (14)

^a^"CMU-C": Universal Health Coverage, the French social security program.

^b^LOS: length of stay.

^c^ICU: intensive care unit.

^d^SAPS II: Simplified Acute Physiology Score II.

^e^NIV: noninvasive ventilation.

### Selection of SOIs, HRU Domains, and Items

SOIs included asthenia, neurological symptoms, symptoms associated with the thoracic region (respiratory and cardiac) and musculoskeletal and gastrointestinal symptoms [6,7,14,22].

Eight HRU domains were chosen to identify health care most likely to be related to SOIs, and hence to PACS: (1) medical visits, (2) technical procedures, (3) dispensed medications, (4) biological analyses, (5) oxygen therapy, (6) rehabilitation, (7) hospital admissions, and (8) emergency and nurse visits (Table 2).

**Table 2:** Domains and specific items of HRU^a^ selected by experts to identify health care resources potentially related to postacute COVID-19 syndrome.

	Domain of HRU	Items of interest
1	Medical visits	General Practitioners; Dermatology; Pneumology; Ear-Nose-Throat; Neurology; Rheumatology; Infection Diseases; Cardiology; Physical medicine and rehabilitation; Psychiatry; Pain Management; Addictology; Hospital Practitioners
2	Technical procedures	Lung Function Tests; X-ray (chest, shoulders, knees); Lower limbs Doppler; ECG^b^; ENMG^c^; EEG^d^; MRI^e^; CT^f^ scan; Peripheral nerves sonography; Nasal endoscopy; Olfactometric tests; Audiogram
3	Dispensed medications	Paracetamol; Antidepressants used for pain management; Antiepileptics used for pain management; WHO^g^ Level II analgesics (weak opioids); Strong opioids; Other analgesics; Vitamin D; Anticoagulants; Beta-blockers; NSAIDs^h^; Antidepressants; Antidepressants without analgesic activity; Anxiolytics; Hypnotics; Inhaled corticosteroids; Short acting bronchodilators; Oral corticosteroids; Chloroquine hydroxychloroquine; Flu vaccine; Midodrine
4	Biological analyses	Full blood count; C-reactive protein; Troponin
5	Oxygen therapy	In- and out hospital
6	Rehabilitation	Swallowing rehabilitation; Vocal rehabilitation; Physiotherapist (out-of-hospital); Occupational therapy; Exercise testing
7	Hospitalizations in post discharge period	Recovery facilities, at home hospitalization, and acute hospital admissions
8	Others	Nurse visits at home, Emergency Room visits

^a^HRU: health care resource use.

^b^ECG: electrocardiogram.

^c^ENMG: electroneuromyogram.

^d^EEG: electroencephalography.

^e^MRI: magnetic resonance imaging.

^f^CT: computed tomography.

^g^WHO: World Health Organization.

^h^NSAID: nonsteroidal anti-inflammatory drug.

### HRU Changes in the Pre– and Post–COVID-19 Periods and PP Ratios

Major changes in HRU were observed for 32 out of 62 (51%) items in all domains, as shown in Tables 3 and 4.

**Table 3:** Changes in health care resource use across all studied domains and items, comparing pre– and post–COVID-19 periods.

HRU^a^ domains and items			Population with at least 1 HRU in the studied period							HRU PP rate ratio (monthly), 95% CI
			Before, n (%)		After, n (%)		PP^b^-Ratio			
**Medical visits**										
	All types	54,948 (79.8)		57,287 (83.2)		1.04		1.2 (1.14-1.26)		
	General practice	49,148 (71.4)		51,014 (74.1)		1.04		1.14 (1.13-1.16)		
	Dermatology	1711 (2.5)		1472 (2.1)		0.86		1 (0.97-1.04)		
	Respiratory care medicine	*559 (0.8)* ^c^		*1347 (2.0)*		*2.41*		0.67 (0.61-0.73)		
	Otolaryngology	744 (1.1)		677 (1.0)		0.91		0.67 (0.61-0.72)		
	Neurology	482 (0.7)		487 (0.7)		1.01		1 (094-1.07)		
	Rheumatology	1187 (1.7)		959 (1.4)		0.81		1 (0.94-1.06)		
	Infection diseases	0 (0)		<10 (<0.01)		N/C^d^		—^e^		
	Cardiology	*2120 (3.1)*		*2658 (3.9)*		*1.25*		1 (0.9-1.11)		
	Physical medicine	146 (0.2)		109 (0.2)		0.75		1.33 (1.08-1.65)		
	Psychiatry	1471 (2.1)		1507 (2.2)		1.02		1 (0.92-1.09)		
	Hospital medicine	*19,439 (28.3)*		*23,451 (34.1)*		*1.21*		1 (0.88-1.14)		
	Pain medicine	35 (<0.1)		30 (<0.1)		0.86		1 (0.72-1.38)		
**Technical acts**										
	All types	*22,112 (32.1)*		*36,373 (52.9)*		*1.64*		1.2 (1.17-1.23)		
	Lung function testing	*3795 (5.5)*		*13,635 (19.8)*		*3.59*		1.33 (1.26-1.41)		
	Radiography	*11,717 (17.0)*		*14,277 (20.7)*		*1.22*		1 (0.96-1.04)		
	Lower limb doppler	491 (0.7)		642 (0.9)		1.31		1 (0.94-1.06)		
	ECG^f^	*9174 (13.3)*		*13095 (19.0)*		*1.43*		1 (0.96-1.04)		
	ENMG^g^	*676 (1.0)*		*1178 (1.7)*		*1.74*		1 (0.92-1.08)		
	EEG^h^	*501 (0.7)*		*740 (1.1)*		*1.48*		1 (0.88-1.14)		
	MRI^i^	*1771 (2.6)*		*2902 (4.2)*		*1.64*		1 (0.97-1.03)		
	CT^j^ scan	*4054 (5.9)*		*18679 (27.1)*		*4.61*		1 (0.98-1.03)		
	Nerves sonography	756 (1.1)		880 (1.3)		1.16		1 (0.92-1,08)		
	Nasal endoscopy	606 (0.9)		713 (1.0)		1.18		1 (0.92-1.09)		
	Olphactometry	0 (0)		<10 (<0.01)		N/C		—		
	Audiogram	1259 (1.8)		1501 (2.2)		1.19		1 (0-94-1.06)		
**Laboratory blood testing**										
	All types	*28,109 (40.8)*		*42,741 (62.1)*		*1.52*		1.33 (1.31-1.36)		
	Full blood count	*27,666 (40.2)*		*36,533 (53.1)*		*1.32*		1.25 (1.23-1.27)		
	CRP^k^	*17,599 (25.6)*		*26,865 (39.0)*		*1.53*		1.33 (1.3-1.37)		
	Troponin	*878 (1.3)*		*1773 (2.6)*		*2.02*		1 (0.9-1.11)		
**Oxygen therapy**										
	All types	*1182 (1.7)*		*3307 (4.8)*		*2.8*		0.89 (0.82-0.96)		
	Outpatient	*608 (0.9)*		*2419 (3.5)*		*3.98*		0.78 (0.73-0.83)		
	Inpatient	*618 (0.9)*		*1019 (1.5)*		*1.65*		1.25 (1.07-1.47)		
**Rehabilitation**										
	All types	*2000 (2.9)*		*5805 (8.4)*		*2.9*		*2 (1.79-2.23)*		
	Swallowing therapy	0 (0)		<10 (<0.01)		N/C		N/C		
	Vocal rehabilitation	0 (0)		<10 (<0.01)		N/C		N/C		
	Outpatient physical therapy	*34 (<0.1)*		*2841 (4.1)*		*83.56*		0.95 (0.69-1.29)		
	Occupational therapy	*822 (1.2)*		*1517 (2.2)*		*1.85*		0.92 (0.81-1.05)		
	Exercise test	*1159 (1.7)*		*1810 (2.6)*		*1.56*		1 (0.96-1.04)		
	Rehabilitation facility	*1946 (2.8)*		*3712 (5.4)*		*1.91*		1 (0.94-1.06)		
	Hospital at home	126 (0.2)		645 (0.9)		5.12		0.8 (0.64-1)		
	New Hospitalization	*13,014 (18.9)*		*19,809 (28.8)*		*1.52*		1.14 (1.08-1.21)		
**Others**										
	Nurses home visits	*24,561 (35.7)*		*33,946 (49.3)*		*1.38*		0.87 (0.85-0.9)		
	Emergency department visits	8049 (11.7)		8827 (12.8)		1.1		1 (0.97-1.04)		

^a^HRU: health care resource use.

^b^PP: post- and pre (after-before, for ratio and rate; log-normalized).

^c^Values of health care resource use with major changes are italicized.

^d^N/C: noncalculated (smallest values or zero denominator).

^e^Not applicable.

^f^ECG: electrocardiogram.

^g^ENMG: electroneuromyogram.

^h^EEG: electroencephalography.

^i^MRI: magnetic resonance imaging.

^j^CT: computed tomography.

^k^CRP: C-reactive protein.

**Table 4:** Changes in health care resource use across medication dispensing, comparing pre– and post–COVID-19 periods.

Items in the HRU^a^ domain of medication dispensing	Population with at least 1 HRU in the studied period				HRU PP rate ratio (monthly) with 95% CI
	Before, n (%)	After, n (%)	PP^b^ ratio		
All types	50,794 (73.8)	52,500 (76.3)	1.03	1.12 (1.1-1,14)	
Acetaminophen	31,813 (46.2)	33,537 (48.7)	1.05	1.1 (1.08-1.12)	
Antidepressants with CPMA^c^	1924 (2.8)	2117 (3.1)	1.1	0.9 (0.85-0.95)	
Antiepileptic with CPMA	*4707 (6.8)* ^d^	*5792 (8.4)*	*1.23*	0.91 (0.88-0.94)	
Weak opioids	9710 (14.1)	9164 (13.3)	0.94	0.88 (0.84-0.93)	
Strong opioids	*1302 (1 9)*	*2262 (3.3)*	*1.74*	0.88 (0.78-0.98)	
Other analgesics	3981 (5.8)	4430 (6.4)	1.11	1.17 (1.06-1.28)	
Vitamin D	*14,494 (21.1)*	*18,337 (26.6)*	*1.27*	1.2 (1.18-1.22)	
Anticoagulants	*8586 (12.5)*	*14,445 (21.0)*	*1.68*	1.08 (1.05-1.12)	
Beta-blockers	14,588 (21.2)	15,919 (23.1)	1.09	1 (098-1.02)	
NSAIDs^e^	12,511 (18.2)	7412 (10.8)	0.59	0.8 (0.78-0.82)	
Antidepressors	10,752 (15.6)	12,376 (18.0)	1.15	0.93 (0.91-0.95)	
Antidepressors without CPMA	9266 (13.5)	10,828 (15.7)	1.17	0.93 (0.91-0.95)	
Anxiolytics	*11,952 (17.4)*	*14,916 (21.7)*	*1.25*	0.92 (0.9-0.95)	
Hypnotics	*5436 (7.9%)*	*6759 (9.8)*	*1.24*	0.93 (0.89-0.97)	
Inhalation corticoids	6402 (9.3%)	7113 (10.3)	1.11	1 (0.97-1.03)	
Bronchodilators	*4546 (6.6%)*	*5574 (8.1)*	*1.23*	0.86 (0.82-0.9)	
Hydroxychloroquine	11 (<0.1)	<10 (<0.01)	0.82	1.5 (0.89-2.54)	
Flu vaccine	*6199 (9.0)*	*9678 (14.1)*	*1.56*	1 (1-1)	
Midodrine	163 (0.2)	209 (0.3)	1.28	1 (0.82-1.22)	

^a^HRU: health care resource use.

^b^PP: post-pre (After-Before, for ratio and rate; log-normalized).

^c^CPMA: chronic pain modulation activity.

^d^Values of health care resource use with major changes are italicized.

^e^NSAID: nonsteroid anti-inflammatory drug.

#### Medical Visits

There was no major change in the percentage of patients with any medical contact (PP ratio=1.04). For specific medical specialties, the most important changes were observed for 1 or more visits to respiratory care physicians (PP ratio=2.41), cardiologists (PP ratio=1.25), and hospital practitioners (PP ratio=1.21). Changes in the rates of visits to neurologists, psychiatrists, pain specialists, and infection diseases specialists were not major.

#### Technical and Diagnostic Procedures

There was a major change in the overall percentage of users of any technical procedure (PP ratio=1.64), with the most important changes for computed tomographic scans (PP ratio=4.61), Lung Function Testing (PP ratio=3.59), electromyography (PP ratio=1.74), magnetic resonance imaging (PP ratio=1.64), electroencephalography (PP ratio=1.48), electrocardiography (PP ratio=1.43), and x-rays (PP ratio=1.22).

#### Biological Analyses

There was a major change in the percentage of patients with any laboratory analysis (PP ratio=1.52). There were major changes in the percentage of patients undergoing troponin dosage (PP ratio=2.02), C-reactive protein dosage (PP ratio=1.53), and full blood count assessment (PP ratio=1.32), while 18,648 patients (27% of the population) had a SARS-CoV-2 polymerase chain reaction test result in the post–COVID-19 period.

#### Oxygen Therapy

There was a major change in the overall percentage of patients using oxygen therapy (PP ratio=2.8), both as outpatients (PP ratio=3.98) and during hospital stays (PP ratio=1.65).

#### Rehabilitation

There was a major change in the overall percentage of patients undergoing rehabilitation (PP ratio=2.90), both as outpatients (PP ratio=83.56) and as inpatients (PP ratio=1.85), and a major change in the percentage of patients undergoing exercise tests (PP ratio=1.56).

#### Recovery Facilities, At-Home Hospitalizations, and Acute Hospital Admissions

There was a major change in the percentage of patients admitted to long-term care facilities (PR ratio=1.91), as well in the percentage of patients hospitalized at home (PP ratio=5.12), and in the percentage of patients hospitalized for acute care (PP ratio=1.52), with major changes in the percentage of patients admitted for chronic pain (PP ratio=1.50), assessment of nononcologic chronic conditions (PP ratio=2.21), congestive heart failure (PP ratio=1.99), and surveillance of dialysis (PP ratio=1.86).

#### Other HRU

Major changes in the percentage of patients visited by nurses were observed (PP ratio=1.38). There was no major change for emergency department visits.

#### Dispensed Medications

There was no major change in the percentage of patients with 1 or more dispensation (PP ratio=1.03), but the rates of medication dispensation increased with a PP ratio of 1.12. Major changes were, however, observed in the percentage of users of strong opioids (PP ratio=1.74), anticoagulants (PP ratio=1.68), flu vaccines (PP ratio=1.56), vitamin D (PP ratio=1.27), anxiolytics (PP ratio=1.25), hypnotics (PP ratio=1.24), antiepileptics with analgesic effects (PP ratio=1.23), and short acting bronchodilators (PP ratio=1.22). The percentage of users of nonsteroidal anti-inflammatory drugs and weak opioids decreased markedly. All details are presented in Table 4.

## Discussion

### Principal Findings

Using the French National Claims Data (SNDS) that record individual HRU, we assessed HRU in the post-discharge period for 68,822 patients hospitalized for COVID-19 infections during the initial wave (>February 2020), aiming to identify new HRU suggestive of PACS. In this frail population (mean age 66 years, common comorbidities), post–COVID-19 HRU increased largely compared to pre–COVID-19 HRU for all domains of care investigated, confirming the major impact of severe COVID-19 infections on the overall health status of affected patients. The overall high HRU, however, prevented the identification of distinct HRU profiles suggestive of PACS, and these descriptive results need to be completed with methods for controlling for confusion bias through subgroup analyses.

Our study population included frail patients, with a mean age of 66 years, and with 3-4 comorbidities (Charlson Comorbidity Index of 3.6), with a large prevalence of comorbidities identified from Chronic Disease Statuses, such as diabetes (23%), cardiac diseases—insufficiency (24%) and coronary diseases (11%), psychiatric disorders (18%) and malignancy (14%), and a high prevalence of CMU-C (Complémentaire santé solidaire [universal health coverage]; 15%) suggestive of social deprivation. More than one-fifth (22.8%) of the initial population deceased during either hospitalization or the early (30 days) postdischarge days, or during a later (months 2-6) postdischarge period, in line with the mortality data observed during the first year of the pandemic, before arrival of vaccines or specific therapy [18].

After defining a list of Symptoms of Interest (SOIs), 8 domains of HRU were considered to be of interest: medical contacts (all specialties), technical procedures, dispensed medications, biological analyses, oxygen therapy, rehabilitation, rehospitalizations, emergency department visits, and nurse visits. The choice was made to use the percentages of patients consuming once or more specific resources among the study population in the pre– and post–COVID-19 periods and the ratios of percentages—post- or prepercentage users—as primary outcomes. Our approach of counting HRU only once per patient, regardless of frequency, may lead to an underestimation of HRU increases. For example, a patient with 1 pre–COVID-19 visit and 10 post–COVID-19 visits would be recorded the same as a patient with 1 visit in each period. While our primary focus was on identifying new health care needs post COVID-19 infection, we acknowledge that this method does not capture the full extent of increased health care use or the intensity of use, which could be clinically relevant, especially for understanding the impact on health care systems. The PP ratio rate we calculated provides additional context for interpreting our findings. The rates of use of any item in the pre– and post–COVID-19 periods would in theory have been an alternative choice, but the organization of care was severely disrupted in the first post–COVID-19 year (2020), with priority devoted to screening and care of the many new COVID-19 cases, and as a rule, the patients diagnosed with COVID-19 underwent therapeutic or diagnostic interventions at distant intervals to prevent additional burden to the health care system. In that context, the identification of any new use of a specific item of HRU mattered more than the rates of use of that item. This approach emphasized that the new use of many items was frequent, most strikingly for pulmonary care physicians, cardiologists, and hospital practitioners, use of computed tomographic scans and magnetic resonance imaging, lung function tests, use of strong opioids, anticoagulants and flu vaccines, anxiolytics and hypnotics, troponin dosage, use of oxygen therapy, in- and outpatient rehabilitations, admissions to recovery facilities, hospitalization at home and acute hospitalizations, and nurse visits.

The findings are in line with evidence underlining high general post–COVID-19 HRU, especially for care associated with respiratory system and cardiology, pain, anxiety and insomnia, coagulation disorders and functional disorders, likely due to the high prevalence of symptoms described after acute COVID-19 infections, such as fatigue, palpitation, dyspnea, headaches, and musculoskeletal pain, reported by longitudinal studies from different countries.

The major increases in the number of users of distinct domains of HRU and the parallel changes in the frequency of the new use of many items likely reflected the severity of the initial infection in this frail population, including its impact on preexisting comorbidities, so preventing an appropriate identification of PACS and its impact on HRU in the early post–COVID-19 period. It, however, identified highly consumed resources, which would permit to allocate resources more efficiently in case of new viral pandemics [4].

When individuals with convalescent COVID-19 have been compared to control individuals who had not been infected, the substantial burden of the postacute COVID-19 sequelae has been demonstrated at 6 months [19]. Particularly high use of health care services has been described in the 30 days after discharge, supporting our decision to exclude this time window from the analyses [4]. Other French data have demonstrated significant increases (from 5% to 25%) in anxiolytics, hypnotics antidepressants, antidiabetics, and antihypertensive drugs dispensed during the first 5 months after the beginning of pandemics in the full French population, but we observed lower figures in our population, likely due to our selection criteria of patients with severe COVID-19 [23].

Our study had several strengths. We identified all hospitalizations for severe COVID-19 that occurred in France during the early phase of the pandemic, and our population may be considered as exhaustive, supporting the validity of our findings [24]. The list of domains and items of interest were defined by experts based on evidence available at the time of study [14], and it optimized the likelihood of collecting data leading to PACS identification. The lack of positive finding provides, however, support to the hypothesis that in our study population of severe COVID-19 with common preexisting comorbidities, PACS lead to complaints that were minor relative to symptoms attributable to COVID-19, the hefty hospital care, and the impacted comorbidities [25]. As the study was performed in the first year of the pandemic, before the arrival of vaccines and specific therapy, the likelihood of observing PACS was, however, optimal, and the absence of specific HRU supports the hypothesis that PACS had a relatively minor impact in the study population compared to the impact of the initial infection, the heavy hospital care, and the worsened comorbidities. The study design, with a direct, specific, and valid, comparison of 2 episodes of care close to each other supports this conclusion.

Some limitations must be acknowledged. Our study included only patients hospitalized for COVID-19, excluding those with milder forms of the disease. The patients with less severe COVID-19 likely had lower post–COVID-19 HRU, which might have helped identify milder symptoms suggestive of PACS. However, our study compared HRU of the same population before and after pandemics, and included cases as their own control, supporting the robustness of our findings. Also, the data were extracted for patients infected during the first wave in France, before major changes were implemented in patients’ care, such as less invasive hospital care, vaccination or specific COVID-19 therapy. For instance, the management of severe forms of COVID-19 during the first wave may be responsible for high prevalence of post–intensive care syndrome, which can be difficult to differentiate from potential PACS [26]. In addition, COVID-19 characteristics changed with time, with successive virus mutations, and development of collective immunity [27]. As already mentioned, one must also consider that the first pandemic wave was associated with considerable changes in health care delivery. In France, there was a substantial decrease in hospitalization rates for several conditions including geriatric [28], stroke [29], access to chronic pain structures [30], and others, because of hospitals’ overflow with patients with COVID-19 and national policies with strong central response restricting local authorities to adopt appropriates postdischarge measures based on the local epidemiological and populational situation during the first pandemic wave [31]. Therefore, the post–COVID-19 HRU might be constrained by the maximum availability of HRU itself, as it has been demonstrated for underrecognized deaths attributable to COVID-19 in the United States [32]. Our method of counting HRU only once per patient may underestimate the true increase in HRU post the COVID-19 emergency. Future research should consider analyzing both initiation and frequency of HRU where data permit, to provide a more comprehensive picture of the post–COVID-19 health care use patterns. Using methods such as clustering could help identify distinct patient groups and postdischarge HRU patterns.

Lastly, the use of the French National Health System claims database does not permit the identification of exhaustive clinical information beyond the diagnosis associated with hospitalization, making the study of PACS, as defined by clinical criteria, challenging.

### Conclusions

Patients discharged from hospital for a severe COVID-19 infection during the first pandemic wave comprised a frail community with high mortality rates and increased levels of post–COVID-19 HRU compared to their pre–COVID-19 HRU, particularly for the care of newly acquired thoracic conditions.

Collected HRU data did not permit the identification of specific patterns of care suggestive of PACS, likely due to the burden of complaints attributable to hospital care and the common prevalence of preexisting comorbidities.

These purely descriptive analysis would merit further analysis in clusters, to verify whether distinct clusters of health care pathways can be identified, including pathways likely suggestive of PACS.

## Appendix

Multimedia Appendix 1 Formulae and SAS script.

## Abbreviations

CMU-C: complémentaire santé solidaire (universal health coverage)
HRU: health care resource use
ICD-10: International Statistical Classification of Diseases, Tenth Revision
PACS: postacute COVID-19 syndrome
PP ratio: post- or pre- ratio
SNDS: Système National des Données de Santé (National Health Data System)
STROBE: Strengthening the Reporting of Observational Studies in Epidemiology
SOI: symptom of interest

## Data Availability

The data used in this research are the property of the French National Health System claims data (SNDS) and are available upon the request formulated to the SNDS.
