# Supplementary material for: Health Care Resource Use and Total Mortality After Hospital Admission for Severe COVID-19 Infections During the Initial Pandemic Wave in France: Descriptive Study
Source: JMIR Public Health Surveill. 2024 Sep 11;10:e56398. doi: 10.2196/56398 (PMC11425017; doi:10.2196/56398)
Supplement: Multimedia Appendix 1 [file publichealth_v10i1e56398_app1.docx]

Appendix : calculation of a log-transformed event post-pre rate ratio along with the 95% confidence intervals.

Let μ for Mean; σ for Standard Deviation; n for Sample Size

1. Log-transformed rates:

log(rate_pre_) = log(μ_pre_)

log(rate_post_) = log(μ_post_)

2. Log-transformed ratio:

log (μ_post_/μ_pre_) = log(μ_post_)-log(μ_pre_)

3. Standard error calculation:


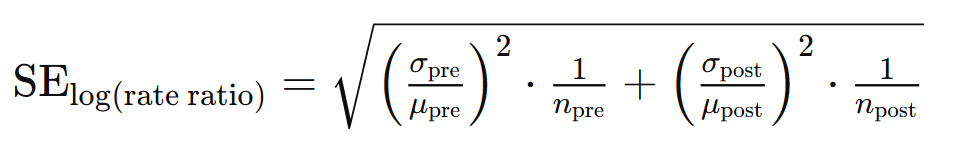


4. Confidence intervals on log scale:


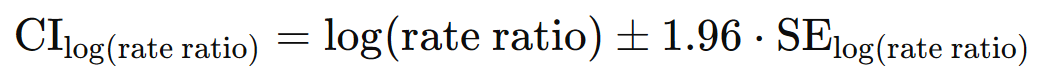


5. Exponentiate to get final rate ratio and CIs:


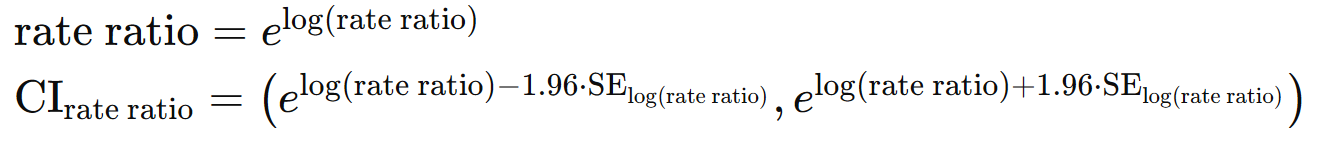


SAS Script

| /* Define the variables */  /*mu_pre - mean pre-exposure rate */  /*sigma_pre - standard deviation pre-exposure rate */  /*n_pre - sample size pre-exposure */  /*mu_post - mean post-exposure rate */  /*sigma_post - standard deviation post-exposure rate */  /*n_post - sample size post-exposure */  /* Calculate log-transformed rates */  %let log_rate_pre = %sysfunc(log(&mu_pre));  %let log_rate_post = %sysfunc(log(&mu_post));  /* Calculate log-transformed ratio */  %let log_rate_ratio = %sysevalf(&log_rate_post - &log_rate_pre);  /* Calculate the standard error of the log-transformed ratio */  %let se_log_rate_ratio = %sysevalf(sqrt(((&sigma_pre / &mu_pre)**2 / &n_pre) + ((&sigma_post / &mu_post)**2 / &n_post)));  /* Calculate the 95% confidence intervals on the log scale */  %let z = 1.96; /* z value for 95% confidence */  %let ci_lower_log = %sysevalf(&log_rate_ratio - &z * &se_log_rate_ratio);  %let ci_upper_log = %sysevalf(&log_rate_ratio + &z * &se_log_rate_ratio);  /* Exponentiate to get the final rate ratio and confidence intervals */  %let rate_ratio = %sysevalf(exp(&log_rate_ratio));  %let ci_lower = %sysevalf(exp(&ci_lower_log));  %let ci_upper = %sysevalf(exp(&ci_upper_log));  /* Display the results */  data results;  log_rate_pre = &log_rate_pre;  log_rate_post = &log_rate_post;  log_rate_ratio = &log_rate_ratio;  se_log_rate_ratio = &se_log_rate_ratio;  ci_lower_log = &ci_lower_log;  ci_upper_log = &ci_upper_log;  rate_ratio = &rate_ratio;  ci_lower = &ci_lower;  ci_upper = &ci_upper;  run;  proc print data=results;  title "Log-Transformed Event Rate Post-Pre Ratio with 95% Confidence Intervals";  run; |
| --- |
